# Polymorphisms of Dopamine Receptor Genes and Parkinson’s Disease: Clinical Relevance and Future Perspectives

**DOI:** 10.3390/ijms22073781

**Published:** 2021-04-06

**Authors:** Luca Magistrelli, Marco Ferrari, Alessia Furgiuele, Anna Vera Milner, Elena Contaldi, Cristoforo Comi, Marco Cosentino, Franca Marino

**Affiliations:** 1 PhD Program in Clinical and Experimental Medicine and Medical Humanities, University of Insubria, 21100 Varese, Italy; magis.luca@gmail.com (L.M.); afurgiuele@uninsubria.it (A.F.); 2 Movement Disorders Centre, Neurology Unit, Department of Translational Medicine, University of Piemonte Orientale, 28100 Novara, Italy; milner.vera@gmail.com (A.V.M.); elena.contaldi@aslvc.piemonte.it (E.C.); 3 Centre of Research in Medical Pharmacology, University of Insubria, 21100 Varese, Italy; marco.ferrari@uninsubria.it (M.F.); marco.cosentino@uninsubria.it (M.C.); franca.marino@uninsubria.it (F.M.); 4 PhD Program in Medical Sciences and Biotechnology, University of Piemonte Orientale, 28100 Novara, Italy; 5 Center of Research in Neuroscience, University of Insubria, 21100 Varese, Italy

**Keywords:** Parkinson’s disease, dopamine receptor, non motor symptoms

## Abstract

Parkinson’s disease (PD) is a neurodegenerative disease caused by loss of dopaminergic neurons in the midbrain. PD is clinically characterized by a variety of motor and nonmotor symptoms, and treatment relies on dopaminergic replacement. Beyond a common pathological hallmark, PD patients may present differences in both clinical progression and response to drug therapy that are partly affected by genetic factors. Despite extensive knowledge on genetic variability of dopaminergic receptors (DR), few studies have addressed their relevance as possible influencers of clinical heterogeneity in PD patients. In this review, we summarized available evidence regarding the role of genetic polymorphisms in DR as possible determinants of PD development, progression and treatment response. Moreover, we examined the role of DR in the modulation of peripheral immunity, in light of the emerging role of the peripheral immune system in PD pathophysiology. A better understanding of all these aspects represents an important step towards the development of precise and personalized disease-modifying therapies for PD.

## 1. Introduction

Parkinson’s disease (PD) is a common neurodegenerative disease, with an overall prevalence of 0.3%, which tends to increase with age [1]. PD is clinically characterized by a wide array of motor and nonmotor manifestations that have a great impact on patients’ quality of life with both social and economic load [2].

In the initial disease stages, when diagnosis is usually established, motor symptoms include slowness of movement (bradykinesia), rest tremor and muscle rigidity. At this stage, patients may also complain of hyposmia, constipation, fatigue, sleep disturbances, mood deflection and impulse control disorder [3] As the disease progresses, motor complications may appear, consisting in wearing-off of the benefit provided by medication or even failure or delay of the medication effect. Involuntary choreiform movements appear when the blood levels of medication either peak or drop. The nonmotor features of this advanced disease stage may consist in visual hallucinations, cognitive decline and autonomic dysfunction (orthostatic hypotension, urinary dysfunction).

The pathological hallmark of PD is represented by the degeneration of the dopaminergic neurons in the pars compacta of the midbrain substantia nigra. Cell loss is accompanied by the accumulation of alpha-synuclein (α-syn) and it is currently believed that α-syn accumulation is correlated with PD progression [4]. Currently, the gold standard treatment for PD consists of dopamine (DA) replacement therapy (DRT) aiming at counterbalancing DA loss caused by nigrostriatal degeneration. Accordingly, the most used drugs for PD treatment are the DA precursor Levodopa, alone or together with MAO-B and COMT inhibitors, and DA-agonists. Unfortunately, as disease progresses, the benefits of symptomatic therapies tend to wear off [5] and to be counterbalanced by the onset of side effects and complications [6]

There are five types of DR, D1, D2, D3, D4 and D5, which are members of the G-protein coupled receptor family [7]. The dopaminergic receptor (DR) subtypes are divided into two families according to their pharmacological profile and second messenger coupling: the “D1-like”, including D1 and D5 which activate adenylate cyclase, and “D2-like” including types D2, D3 and D4 which inhibit adenylate cyclase [8]. The final effect of D1-like activation (D1 and D5) can be both excitation (via opening of sodium channels) and inhibition (via opening of potassium channels), while the ultimate effect of D2-like activation (D2, D3 and D4) is inhibition of target neuron [9].

D1 receptors are the most abundant DR in the human nervous system followed by D2 and other DR (D3, D4 and D5) whose levels are significantly lower [9].

Despite the key role of dopaminergic pathways in the pathogenesis as well as in the pharmacotherapy of PD, evidence on the role of genetic polymorphisms in DR and related pathways is still fragmentary. In this regard, several functional single nucleotide polymorphisms (SNPs, i.e., DNA sequence variations occurring when a single nucleotide in the genome differs between paired chromosomes), the most common type of polymorphisms in the human genome, have been identified in dopamine receptor genes (DR) [10,11]. Among these SNPs, some have been related with other neuropsychiatric conditions such as schizophrenia [12,13], attention deficit hyperactivity disorder [14,15], addictions [16] and even to clinical aspects of PD [17,18].

Furthermore, DR play a key role in the regulation of peripheral immunity [19,20,21] and recent findings suggest a role for the peripheral immune response in PD. For example, it has been shown that α-syn is recognized by T cells, thus suggesting a relationship between protein deposition, neuronal loss and immune response [4,22]. Since immune cells express DR, it is reasonable to suppose that SNPs in genes coding for these receptors could modulate functions of these cells. In this regard, it has been recently suggested that SNPs in DRs could influence immune cell functions in different ways [23,24]. Nonetheless, the relevance of such effects on PD development and progression, including response to therapy, has never been examined so far.

In the present review, we collected and critically appraised available evidence on the role of genetic polymorphisms in dopaminergic receptors with respect to: (i) risk of PD development, (ii) variability of PD phenotypes, (iii) response to pharmacological therapy and risk of side effect, (iv) immune function.

## 2. Pharmacogenetics of Dopaminergic Receptors

Several genetic polymorphisms have been described in DRs, and it had been suggested that *DR*s variability may be involved in both disease susceptibility and individual response to pharmacological treatments in PD.

### 2.1. Genetic Polymorphisms in Dopamine Receptor D1 Gene

The DR D1 gene (*DRD1*) contains two exons [25] and its alternative splicing results in the expression of two different transcripts. The genetic analysis of the *DRD1* sequence has allowed the identification of various polymorphisms located within the gene whose functional consequences are still not fully understood. Among the most studied SNPs, rs686 contains a G-to-A substitution, with a frequency of the minor A allele of about 60% in Caucasian population. This SNP was associated with increased promoter activity and higher *DRD1* expression. Another important SNP in *DRD1* is rs5327, consisting in an A-to-C substitution (amino acid changing Thr37Pro), which was associated with reduction in total and surface receptor expression [26], while rs1799914, a G-to-A substitution, featured reduction in both mRNA expression levels and Bmax. This SNP was also associated with a reduced heterodimerisation of the D2 receptor [27].

Another important SNP in *DRD1* is A-to-G substitution at position −48 in promoter region (rs4532); this SNP shown a frequency of 30% in Caucasian population, whereas for several other *DRD1* SNPs, such as rs5330, rs5331, rs13306309, no biological or clinical functions have been described yet.

### 2.2. Genetic Polymorphisms in Dopamine Receptor D2 Gene

The DR D2 gene (*DRD2*) is located on chromosome 11q23 and contains six introns. Through the alternative splicing, two main variants are generated, named D2S and D2L [28].

More than 200 polymorphisms have been identified in *DRD2*, mostly in the introns and in downstream flanking region [29], which have been associated with addictions (alcohol, cocaine, nicotine and opioid), mood disorders, schizophrenia, movement disorders and drug response (reviewed in [30,31]).

rs1800497 (also known as TAQ1 A), the most studied *DRD2* polymorphism, consists of a C-to-T substitution (amino acid changing Glu713Lys), with a frequency of the minor T allele of about 22% in Caucasian population. This variation was associated with a 40% reduction in D2 receptors expression in the striatum, without affecting receptor affinity [32,33,34,35].

A second important polymorphism in *DRD2* is −141 C Ins/Del (rs1799732). This SNP is located in the promoter region of the *DRD2* with allele frequency of about 9% of the Caucasian population [36] and is associated with a higher DR density in striatum [34].

Another frequent SNP in *DRD2* is rs6277 which consists of a C-to-T substitution at positions 957 located in exon 7. The minor allele (T) has a frequency of about 50% in Caucasian population. The SNP was associated with decreased mRNA stability and translation, reduced DA-induced up-regulation of DR D2 membrane expression in vitro [37], and lower DR D2 expression in cortex and striatum of healthy subjects [38,39,40].

SNPs rs2283265 (consisting of a C-to-G substitution) and rs1076560 (consisting of a G-to-T substitution) alter mRNA splicing and transcription process in exon 6 leading to two isoforms of DRD2, which are D2 long and D2 short and were associated with low mRNA expression levels [31,41,42,43,44]. More specifically, these SNPs were associated with reduced DRD2 expression in prefrontal cortex and striatum as well as with altered activity of the striato-thalamic-prefrontal pathway in healthy subjects [31,45] and in schizophrenia [41]. Two other SNPs were associated with reductions in DRD2 gene activity, in particular rs1801028 (consisting of a C-to-G substitution, amino acid changing Ser311Cys) was associated with reduced DA affinity [46], and rs1079597 (consisting of a G-to-A substitution) with lower receptor binding [32,34]. On the contrary, SNP rs12364283 (consisting of a T-to-C substitution) was associated with increase in DRD2 receptor activity through an increase in mRNA expression levels [47].

Finally, rs2734849, a G-to-A transition, produces an amino-acid change (arginine to histidine) in C-terminal ankyrin repeat domain of ANKK1. Using the luciferase reporter assay, it has been demonstrated that this SNP alters expression level of NF-kB-regulated genes. Since *DRD2* expression is regulated by transcription factor NF-kB, it has been postulated that rs2734849 may indirectly affect DRD2 density, although this hypothesis has not yet been confirmed in ad-hoc study [48].

### 2.3. Genetic Polymorphisms in Dopamine Receptor D3 Gene

DR D3 gene (*DRD3*) contains six exons and expresses five alternative mRNAs [49]. Among *DRD3* polymorphisms, one of the most studied is located in the region coding for the receptor’s amino-terminal extracellular domain and consists in a G-to-A substitution at position 25 (rs6280) (amino acid changing Ser9Gly). This SNP has a frequency of about 40% in Caucasian population. G (Ser9) allele is referred to as allele 1, and A (Gly9) allele as allele 2. AA homozygosis confers a higher DA receptor binding affinity [50].

A clinical role was found also for rs6280 and rs1800828 SNPs [51], while neither a biological or a clinical role for the remainder SNPs in DRD3 (rs963468, rs2134655, rs9817063, rs320435, rs11721264, rs1800828, rs3773678, rs167770, rs167771, rs7633291 and rs2134655) has been identified so far.

### 2.4. Genetic Polymorphisms in Dopamine Receptor D4 Gene

DR D4 gene (*DRD4*) contains four exons. Exon 3, which encodes a cytoplasmic region of the receptor, contains a polymorphism characterized by a varying number of 48-bp repeats VNTR (variable number of tandem repeats) [52]. Nineteen different 48-bp VNTR in twenty-five different haplotypes, encoding for D4 receptors with eighteen different amino acid sequences, have been identified. Alleles with two, four and seven repetitions are the most frequently represented and may influence binding capacity to certain ligands. Besides the VNTR, a 120 bp functional duplication in the 5′ regulatory region was associated with increased transcription factor binding [53] and with reduced promoter activity for *DRD4* [11,54].

Another important SNP in *DRD4* is rs1800443, consisting in a T-to-G substitution (amino acid changing Val194Gly), which was associated with reduction in sensitivity to DA, absence of receptors in the high-affinity state [55] and reduced spiperone binding affinity [56]. rs1800955 consists of a T-to-C change and was associated with greater promoter activity, [57] although this result was not replicated in other studies [54,58,59]. Finally, rs747302, a SNP that consists of a C-at-G substitution at position 62 was associated with longer time to develop visual hallucinations in PD patients [18].

### 2.5. Genetic Polymorphisms in Dopamine Receptor D5 Gene

Genetic polymorphisms’ presence in DR D5 gene (*DRD5*) has been less extensively studied. Some SNPs in *DRD5*, including rs77434021, rs2076907, rs6283 and rs1800762, were located in both coding regions and regulatory regions, the latter including the promoter region and 5′ UTR, which may be involved in the regulation of gene expression [60]. Additionally, a G-to-T substitution (named rs6824806) has been associated with *DRD5* mRNA abundance.

Finally, from a clinical point of view, only the (CA)n dinucleotide repeat, a SNP closely linked to the *DRD5,* has been associated with a significant risk for ADHD [61,62,63].

## 3. Genetic Polymorphisms in Dr and Parkinson’s Disease Risk

Genetic variation of D1-like receptor genes (rs5330, rs5331, rs13306309, rs4532) was studied in association with PD risk, but results were so far inconclusive [63]. As regards D2-like receptor genes, the role of rs1800497 (Taq1A) SNP in *DRD2* on PD risk has been evaluated in several studies. McGuire et al., in a large cohort of more than 1000 patients and controls, detected significant differences among patients with different ethnic origins. In particular, non-Hispanic patients with rs1800497 homozygosis had a 1.5-fold increased risk of PD, while Afro-American patients carrying one rs1800497 allele had a 80–90% PD risk reduction. Moreover, these subjects presented a significant positive association with other *DRD2* SNPs, such as rs6279 and −141 CIns/Del. Authors also hypothesized that these SNPs may be in linkage disequilibrium with other causative polymorphisms [64]. Some other studies support the positive associations between rs1800497 SNP and PD risk [65,66], however, other works do not confirm this [67,68,69,70,71,72].

Another SNP in *DRD2* associated with PD risk was an intronic dinucleotide repeat; in particular, Planté-Bordeneuve showed that patients carrying a 122 bp repeat allele had an increased risk of developing PD [73]. On the contrary, other SNPs in *DRD2* (such as rs1076563, rs6279, rs6278, rs273482, rs1799732, rs17294542, rs1800498, rs2234689) did not show a significant association with PD development [74].

The role of rs6280 SNP in *DRD3* in PD risk is still controversial, since association with earlier PD onset was detected by one study, but not confirmed by another [75,76].

A 5′ UTR 120 bp duplication polymorphism in *DRD4* was reported to influence PD risk in a cohort of Indian subjects. In particular, the 120-bp duplicated allele was protective, while the presence of only one duplicated allele predisposed to PD. When comparing patients from North and South India, the latter presented a positive association with another SNP (rs1800955) in *DRD4* but, due to absence of concordant reports, no definite conclusion was stated [63]. Finally, Kronenberg analyzed a 48-bp VNTR at the third exon of the *DRD4* and found an increased frequency of 4/4 genotype (that is four 48-bp VNTR on both alleles) in patients than controls, even if this difference was not statistically significant [77].

## 4. Genetic Polymorphisms in DR and Parkinson’s Disease Symptoms

### 4.1. PD Motor Symptoms

The role of *DRs* variation in influencing motor features of PD has been poorly studied. There is a German study on a population of 591 PD patients, exploring rs6280 SNP in *DRD3,* a variation previously associated with essential tremor (ET), in which no significant association between the SNP and PD tremor was detected [78,79]. As regards rigidity, there is only one report on 126 African-Caribbean patients showing a positive association with −141 C Ins/Del SNPs in *DRD2*, even though it was referred to antipsychotic-induced parkinsonism and not idiopathic PD [80].

### 4.2. Gastrointestinal Symptoms

As highlighted by the PRIAMO study, almost all PD patients suffer from gastrointestinal (GI) symptoms such as drooling of saliva, difficulty in swallowing, constipation and nausea/vomiting [81]. Some of these symptoms, like constipation, may even precede motor manifestations by several years, whereas others, like nausea and vomiting, occur early in the disease course and may represent adverse events of the pharmacological therapy [82]. GI functions are controlled by the central, autonomic and enteric nervous system, and the involvement of dopaminergic neurons has been recognized. Thus, dopaminergic neuronal loss and Lewy bodies deposition are probably involved in impaired gastric emptying that leads to GI symptoms [83,84].

Rieck et al. investigated the relationship between rs1799732 SNP in *DRD2* and rs6280 SNP in *DRD3* and GI symptoms induced by levodopa treatment in a group of 217 PD patients with (25.8%) or without (74.2%) GI symptoms. The authors found that both SNPs were associated with GI symptoms occurrence during levodopa therapy and that their effects were independent and probably additive [85]. Since DRD2 and DRD3 are widely distributed in the area postrema and in the gastrointestinal tract, it is conceivable that SNPs in such receptor genes influence their density and DA affinity and consequently contribute to GI symptom development [84,85].

### 4.3. Impulse Control Disorders

Impulse Control Disorders (ICDs) are a category of behavioral disorders characterized by a failure to resist a temptation, urge or impulse repetitively, excessively and compulsively, despite personal and relational consequences [86]. ICDs include pathological gambling, compulsive buying, binge eating, hypersexuality and other impulsive-compulsive behaviors (ICBs) such as hobbyism, punding and the Dopamine Dysregulation Syndrome (DDS) [86]. The pathophysiology of ICDs is not yet completely understood. Different studies suggest that they may be related to neurotransmitter dysfunctions, including DA, norepinephrine and serotonin. In particular, there is great interest in DA networks [86,87,88], indeed, different studies dealing with brain activity provided evidence of alterations in ventral striatum, orbito-frontal cortex and anterior cingulate cortex functioning [89]. Several risk factors for developing ICDs have been identified, including personality traits of high novelty-seeking or of impulsiveness, depression, male sex, substance abuse, younger age, younger age of PD onset, pre-PD history of ICDs, current cigarette smoking, family history of substance abuse or of ICDs, preserved executive functions, higher aggressiveness, irritability, disinhibition and eating disorders [90]. Nevertheless, the main risk factor remains DRT. ICDs affect up to 17.7% of patients with PD on DRT, whereas the prevalence of ICDs in untreated PD patients is similar to that of the general population. However, since not all patients develop this side effect, there should be individual genetic susceptibility.

For this reason, many studies have pointed out an association between genetic polymorphisms in *DR* and ICD.

The role of genetic polymorphisms in *DRD1* in ICDs is controversial. Accordingly, while Zainal Abidin showed that SNPs rs4867798, rs4532 and rs1800497 in *DRD1* were associated with an increased ICDs risk among PD patients in Malaysia [91], another two studies failed to find any association [92,93].

Moving to *DRD3,* Lee studied 404 Korean PD patients and 559 Korean controls and found that TT genotype of rs6280 SNP in *DRD3* was independently associated with the occurrence of ICD [94]. This result is in agreement with a prospective, case-control study of Krishnamoorthy, which showed an independent association between rs6280 in *DRD3* and ICDs in a cohort of 170 Indian PD patients (70 with and 100 without ICDs) and 285 healthy volunteers [95]. Finally, Castro-Martinez associated rs6280 and ICD in a sample of Spanish PD patients with an early onset of the disease (defined by an age at onset of less than 45 years old) [96]. Altogether, these data suggest that the strongest genetic risk factor for ICD development in PD patients may be rs6280. This is not surprising, as DRD3 is mainly expressed in the limbic system and it is thought to act as an auto-receptor in the ventral tegmental area, providing presynaptic negative feedback for regulating extracellular DA level. This polymorphism confers a different receptor affinity, thus impairing the reward–risk assessment in the mesolimbic system [95,96].

Finally, no correlation between SNPs in *DRD2* [91,94], *DRD4* and *DRD5* and ICD has, so far, been found.

### 4.4. Visual Hallucinations

Visual hallucinations (VHs) are abnormal perceptions without a physical stimulus and they represent the most common psychotic symptoms in PD patients [97]. Moreover, VHs is associated with later onset of dementia, increased caregiver burden, nursing home placement and increased mortality [98,99]. Prevalence of VHs increases over time, reaching about 60% after 10 years [100] and 75% after 20 years from disease onset [98]. Moreover, even though VHs are predominant in PD patients, the prevalence of non-visual hallucinations (such as tactile, auditory and olfactory ones) shows a similar positive trend: in a longitudinal study, almost 70% of patients experienced multidomain (visual plus nonvisual) hallucinations by 10 years [101].

Risk factors for developing VHs are cognitive impairment, depression, motor symptoms severity, axial impairment, sleep disruption, rapid eye movement behavior sleep disorder (RBD) and visual disturbances [97,98,99]. Regarding pharmacological therapy, an increased risk of VHs was found in PD patients treated with dopaminergic agents, such as rotigotine and rasagiline. However, anti-parkinsonian drugs, although they may worsen VHs, do not represent the main causative agents for this phenomenon [97].

Nevertheless, not all PD patients on dopaminergic therapy develop VHs, thus indicating a suitable genetic predisposition. So far, only few studies have investigated the association between genetic polymorphisms in DR genes and VH in PD patients, with inconclusive results.

Goetz did not find any association between SNPs in *DRD1*, *DRD2*, *DRD3* and *DRD4* and VHs, but only a trend toward over-representation of the DRD3 in patients with VHs [101]. Moreover, while Wang did not detect differences in rs1800497 (*DRD2)*, rs6280 (*DRD3)* and rs6283 (*DRD5)* frequencies in 90 Chinese PD patients with and without hallucinations [102], different results were detected in Caucasian patients. Particularly, Makoff studied the frequencies of two SNPs in *DRD2* (−141 C/del, and rs1800497) and one in *DRD3* (rs6280) in 155 PD patients (84 with and 71 without hallucinations). Authors found that rs1800497 SNP was associated with late-onset hallucinations [103]. Our research group demonstrated that VHs risk was more than 10 times higher in patients carrying rs686, but not rs18100497, SNP in *DRD1*. Moreover, we have found that patients carrying rs4532 SNP had significantly shorter time to VHs, whereas longer time to VHs was found in subjects carrying rs747302 in *DRD4* [18].

The discrepancy among these studies may have different causes. First, different definitions of VHs were used. For instance, Ferrari considered only VHs requiring medical intervention [18], whereas Makoff [103] and Wang [102] considered any VHs, regardless of their severity. Second, ethnic genetic variability may be involved in the different findings between Chinese and Caucasian PD patients. Third, it is possible that other genes and neurotransmitter pathways may be involved in the pathophysiology of VHs and psychosis in PD [104].

### 4.5. Cognitive Decline

Cognitive decline is one of the most common nonmotor symptoms in PD, ranging from mild cognitive impairment (MCI) to dementia [105]. MCI can be present from early stages of the disease, even in 15–20% of de novo patients and there is a cumulative likelihood of developing dementia with disease progression [106]. Approximately 10% of patients present dementia within 3 years of diagnosis, and this value increases up to 46% after 10 years and 83% after 20 years [107]. Cognitive impairment in PD is characterized by executive deficits in planning, set-shifting abilities and working memory and reflects the dopaminergic loss in the dorsal striatum. This area shows connections with the dorsolateral regions of the prefrontal cortex, involved in many executive functions, whose blood flow is modulated by dopaminergic therapy [108,109]. Since not all patients develop cognitive decline, a genetic predisposition may be taken into account.

Bäckström and colleagues investigated whether rs6277 in *DRD2* can affect the development of cognitive deficits in a group of 134 PD patients prospectively followed for 6–10 years. The authors found that the T/T allele in rs6277 SNP in *DRD2* was associated with an increased risk of cognitive decline in PD. Specifically, this genotype significantly increased the hazard for developing cognitive decline 3.2 times in comparison with PD patients with other *DRD2* genotypes, and determined poorer performances in episodic memory and attention [110].

### 4.6. Sleep Attacks

Sleep attacks (SA) are defined as events of overwhelming sleepiness, sometimes preceded by warning symptoms [111]. SA typically occur during different activities of daily living (i.e., eating, writing or driving a car) and last from seconds to minutes [112]. They represent a relatively common complaint among PD patients taking dopaminergic therapy, reaching a prevalence of 13%, regardless of the ethnic group, and up to 66% of the patients had daily episodes. Currently, known risk factors for SA are older age, male sex, longer disease duration, previous report of sleep disturbances, high Epworth Sleepiness Score (ESS) and dopaminergic therapy [112,113].

The role of dopaminergic transmission in sleep modulation is well recognized: dopaminergic neurons are interposed on the sleep circuit [114] and SA are more frequent in patients treated with DA agonists, particularly pramipexole (a D3 agonist), followed by ropinirole (a D2/D3 agonist) and pergolide (a D1/D2 agonist) and SA improve when dopaminergic treatments are reduced or interrupted [113]. However, association with dopaminergic treatment is still controversial, since temporal association and dose–effect relationship have not yet been fully established [115].

Since non-ergoline DA agonists have scarce affinity to the DA D_1_-like receptors, it has been hypothesized that alterations in dopaminergic expression mostly involve D2 receptor family. In this context, Rissling and colleagues explored the frequency of the most common SNPs in D_2_-like receptors genes in a cohort of 274 PD patients, stratified in two groups based on the presence or absence of sudden onset of sleep. The authors found a significant association between the rs1800497 SNP and sudden onset of sleep [116]. Of note, DRD2 plays as a presynaptic autoreceptor in the mesocorticolimbic system whose activation, therefore, might lessen the dopaminergic tone in this system, contributing to the development of daytime sleepiness. By contrast, Paus and colleagues examined 204 PD patients (102 with sleep attacks matched to 102 without sleep attacks, with the same dopaminergic treatment) and did not find any significant association between sleep attacks and 141 C del/ins, rs1800497 SNPs both in *DRD2*, and rs6280 SNP in *DRD3*. However, the authors found higher frequency of homozygosis of 48-bp VNTR with two repeats in *DRD4* among patients with sleep attacks without warning signs, concluding that this allele may represent a risk factor for sleep attacks in PD patients on dopaminergic drugs [117]. These data are summarized in Table 1

## 5. Genetic Polymorphisms in Dr Genes and Response to Antiparkinsonian Dopaminergic Treatment

Treatment of PD is mainly based on levodopa and DA agonists, which determine restoration of basal ganglia circuitry. However, treatment may determine the development of both motor and non-motor symptoms fluctuations [131]. As shown also in previous sections of this review, several genetic polymorphisms in *DR* may cause a change in density and dopaminergic activity in the CSF [132]. Therefore, one may speculate that the demand for dopaminergic medication may be genetically influenced.

On this basis, Paus and colleagues tested whether rs1800497 SNP in *DRD2* receptor was associated with dopaminergic treatment in a population of 503 PD patients [133]. Since no significant differences between this SNP and therapy request were detected, the authors concluded that this variation does not influence dopaminergic requirement in PD patients. Additionally, Xu and co-workers examined the impact exerted by genetic polymorphisms in *DRD2* and *DRD3* on daily doses of DA agonists. In particular, they investigated the association between dinucleotide short tandem repeat (Can-STR) in *DRD2* and rs6280 SNPs in *DRD3* and different doses of DA agonists in 168 PD patients and found that patients carrying rs6280 needed higher doses of pramipexole for effective treatment [134]. This result was confirmed by another study reporting a correlation between rs6280 and response to pramipexole [135].

Concerning the efficacy of anti-parkinsonian therapy, both rs1800497 in *DRD2* and rs6280 in *DRD3* have been correlated with clinical efficacy of pramipexole. Particularly, Liu and colleagues evaluated the response rates (defined as an improvement of at least 20% of the total Unified Parkinson’s Disease Rating Scale—UPDRS score) 2 months after the introduction of pramipexole in a group of 30 Chinese PD patients. The authors found that responses were significantly higher in patients homozygous for rs6280 SNP, whereas no significant association was found for rs1800497 [135].

In the ADAGIO trial, which was designed to assess the impact of rasagiline on disease progression in a large cohort (*n* = 692) of PD patients, rs2283265 and rs1076560 SNPs in DRD2 were associated with a significant improvement of the UPDRS score [136]. Nonetheless, a post hoc analysis of the same trial did detect a statistically significant change of UPDRS score in the delayed phase from week 36 post rasagiline therapy [137].

In a study designed to assess the impact of rs1076560 in *DRD2* on motor task performance in PD patients taking L-DOPA, treatment-induced improvements were only observed in carriers of allele T, which is associated with lower D2 receptor availability, whereas patients’ homozygotes at allele G showed no performance change [138].

Genetic polymorphisms in DR genes have also been investigated with respect to therapy discontinuation. In this regard, Arbouw et al. analyzed a population of 90 PD patients treated with ropinirole or pramipexole. The authors studied 141 C Ins/Del, CA(n) STR, rs1800497 in *DRD2* and rs4646996 in *DRD3* and found that the absence of a 15-repetition CA(n) repeat allele in *DRD2* was associated with a lower rate, while rs4646996 in *DRD3* was associated with a significantly higher rate of discontinuation [139].

Finally, regarding the side effects induced by dopaminergic agents, Dos Santos found that PD patients treated with dopamine agonist and carrying rs6280 SNPs showed an increased risk of developing VHs [129].

### Levodopa-Induced Dyskinesia

Levodopa administration is associated with the development of motor complications, such as motor fluctuations and Levodopa-induced dyskinesias (LIDs) [140]. By definition, motor fluctuation is a phenomenon characterized by the worsening or reappearance of motor symptoms resulting in an “off” state, usually related to low levodopa serum levels [141]. Based on clinic-pharmacological observations, different patterns of levodopa-related motor fluctuations have been described as wearing- off (which is the most common), delayed-ON, no-ON, random ON–OFF [142]. LIDs include different hyperkinetic involuntary movements (most commonly chorea and dystonia), which occur most frequently when levodopa level is high in the plasma. In particular, patients develop limb chorea and/or cranio-cervical dystonia in ON periods and painful foot dystonia during the OFF ones. Finally, diphasic dyskinesia appears immediately after and before a single dose of levodopa and comprises phasic as well as dystonic movements [143].

Levodopa dose (but not its timing of initiation) is a major risk factor for LIDs, together with younger age at PD onset, female sex and lower body weight [144,145]. Moreover, profound inter-individual heterogeneity was found in LID, thus, it is safe to assume that genetic predisposition may play a relevant role in LID appearance. However, so far, the studies focusing on genetic differences in LID lead to conflicting results.

Regarding D1-like DR, no significant association was found between genetic polymorphisms in *DRD1* and *DRD5* and motor complications. In particular, two case-control studies detected no correlation between SNPs in *DRD1* and *DRD5* and LIDs [17,119]. Nonetheless, Dos Santos suggested that rs4532 SNP in *DRD1*, but not rs1800497 in *DRD2*, may play a protector role in the occurrence of LIDs in Brazilian PD patients, by modifying DRD1 expression levels [130].

Moving to D2-like DR, Oliveri investigated whether an intronic CA(n) short tandem repeat (STR) in *DRD2* was associated with peak-dose dyskinesia in a cohort of 98 Italian PD patients. The authors concluded that 13 repetitions and 14 repetitions were more expressed in non-dyskinetic patients who presented 72% risk reduction of developing peak-dose dyskinesia [119]. On the contrary, Strong found that the 14 repetitions and/or the 14/15 repetitions are a risk factor for dyskinesia development [123]. Some differences in the study design might account for these contradictory results. For example, Oliveri detected directly peak-dose dyskinesia during an acute levodopa test, while Strong relied on patient records, including all types of dyskinesias. Moreover, Oliveri compared PD patients with and without dyskinesia, whereas Strong enrolled patients with early- and late-onset dyskinesia.

Some other SNPs in *DRD2* were associated with LIDs. Redenšek found that rs1799732 was associated with LIDs time occurrence after levodopa treatment [128]. Moreover, in 2001, Wang performed a case-control study, involving 40 matched pairs of Chinese PD patients, and found that rs1800497 SNP was significantly more expressed among fluctuating patients [120]. Rieck suggested that patients carrying rs2283265, rs1076560 and rs1800497 and SNPs had significantly higher LID risk [126]. Finally, Zappia showed that 13,14 CA repeats in DRD2 CAn-STR SNP was associated with decreased risk in peak-dose dyskinesia [146]. However, even for correlations between dyskinesia and SNPs in DRD2, reported results in literature are not without contradictions. For example, unlike the above reported results, in some other case-control studies, no significant association was identified between rs1800497 and rs6277 SNPs and LID or motor fluctuations [17,122,125,127,130]

Moving to *DRD3*, neither Wang [120] nor Kaiser [122] found any evidence of correlations between genetic polymorphisms in this gene and dyskinesia. This result was confirmed by Paus, who investigated the impact of rs6280 SNP in *DRD3* on motor complications development in a cohort of 591 patients included in the gene bank of the German Competence Network on Parkinson’s disease. The authors did not detect any association between this SNP and any kind of LIDs [124]. By contrast, Lee [125] and Comi [17] described an association between rs6280 SNP in *DRD3* and LIDs. Finally, Jee-Young Lee [147] found that rs6280 in *DRD3* was associated with diphasic dyskinesia. Since rs6280 SNP is associated with a weaker binding affinity to DA, it has been suggested that SNPs in *DRD3* may induce LID by modifying the sensitization process of the basal ganglia circuitry.

Regarding *DRD4* Kaiser [122] failed to correlate any SNP in this gene with dyskinesia and/or on-off and wearing-off phenomena development and Comi et al. [17] confirmed this negative result.

Table 2 summarises the studies analysing correlations between SNPs in DA receptor and response to pharmacological treatment.

## 6. Role of Dr Genetic Polymorphisms in Peripheral Immunity: Possible Relevance for Pd

Besides a more “direct” action in determining clinical progression of PD and drugs response, DR SNPs may also play an indirect role by modulating the peripheral immune response, which is involved in the pathophysiology of PD [19,20,21], therefore representing a potential therapeutic target for disease modification [152].

DA is a crucial transmitter in the neuro-immune network, and dopaminergic pathways have received increasing interest in the study of adaptive immunity. DA is able to modulate activity of several immune cell subpopulations such as: T and B cells, dendritic cells, macrophages, microglia, neutrophils as well as NK cells (reviewed in [20,153]). Moreover, immune cells express all DRs, with a higher expression of D1-like receptors. Particularly, different cell sub-populations may express different receptors patterns: D1-like receptors are more represented in naïve T cells, while D2-like receptors are more expressed in memory cells [23]. Among immune cells, CD4+ T cells are specifically affected by DA, which subserve an inhibitory loop in human CD4+ CD25 high regulatory T lymphocytes. This specialized T cell subset plays a key role in the control of immune homeostasis [154] and mediates the influence exerted by dendritic cells on the differentiation of naive CD4+ T cells. In PD animal models, these regulatory cells (Treg) are able to modulate microglia differentiation and therefore influence the neurodegenerative process. Particularly, they can modulate microglia proteomics, reducing the expression of protein involved, among others, in cell metabolism and migration and protein degradation [155], and attenuate the Th17-mediated dopaminergic neuronal loss [156]. Furthermore, Treg are reduced in 6-OHDA mice model of PD and this reduction correlates with the central change in microglia profile toward a pro-inflammatory one [157].

PD patients present changes in peripheral cells expression: different studies reported a lower lymphocyte count related to a reduced number of helper CD4+ T cells and B cells [158,159]. Recently, it has been demonstrated that in a large cohort of UK subjects, a lower lymphocyte number represents an important risk factor for a subsequent diagnosis of PD [160]. Additionally, it has recently been suggested that *DR* SNPs could influence immune cell functions in different ways. rs4532 and rs686 in *DRD1* and rs6283 in *DRD5*, alone and in combination, were associated with total count of lymphocytes, as well as CD3+ and CD4+ T lymphocytes, thus indicating a prevalent functional activity of D1-like receptors on these cells [23]. Moreover, SNPs in *DR* including rs4523 affect Treg-induced decrease of Teff cell proliferation in healthy controls [24].

PD patients display a pro-inflammatory peripheral immune phenotype, with a production of cytokines leading to Th1 differentiation [22]. Moreover, presence of circulating auto-antibodies and T cell infiltration in CNS were found in PD subjects [161]. It was suggested that infiltration and reactivation of T cells can prime microglia into a pro-inflammatory phenotype, which can in turn trigger a further detrimental response in the CNS, thus perpetuating the ongoing neurodegenerative process of PD [19]. More recently, experimental work provided evidence on the involvement of immune dysfunction in the development of both motor and non-motor symptoms of PD, including dyskinesia [162], RBD [163] and cognitive decline [164].

Altogether, these findings underline that polymorphisms in *DR* would likely play a role in the immune crosstalk of PD.

Although preliminary in the field of neuro-immunomodulation in PD, the above reported examples stress the importance of the possible role of genetic differences in dopaminergic modulation of immune systems in PD. In our opinion, future investigations in this field may provide a better understanding of PD pathophysiology, and eventually help the identification of new therapeutic targets for this disease [165]

## 7. Conclusions

All these data highlight the importance of personal genetic predisposition in the pathophysiology of PD.

Particularly, *DR* SNPs may be involved not only in disease development, but also in motor and non-motor complications (dyskinesia, visual hallucinations, ICD and cognitive decline), as well as in pharmacological response and side effects induced by dopaminergic agents. Furthermore, they may modulate peripheral cells expression contributing to the creation of an impaired peripheral immunity system, which is known to play a crucial role in the pathophysiology of PD [166].

DRD2 and DRD3 SNPs represent the most promising DR genetic variations in terms of biomarkers identification. Among them, DRD2 rs1800497 and DRD3 rs6280 should be tested in large cohorts of PD patients in order to better clarify their contribution in disease progression.

The evaluation of the relationship between PD progression, response to antiparkinsonian drugs and patients’ genetic profile could be useful in clinical practice since it can help in determining biomarkers for disease evolution at the time of diagnosis and personal response to pharmacological treatment. This approach will be determinant in the creation of a causative and tailored pharmacological approach which, in addition to providing benefits for patients, would reduce the management costs of therapy.

## Figures and Tables

**Table 1:** Studies concerning effects of single nucleotide polymorphisms (SNPs) in dopaminergic receptors (DR) in Parkinson’s disease (PD) patients.

Study Population	Considered SNPs	Main Results	Reference
**Parkinson Disease (PD) Risk**			
100 PD patients and 100 healthy controls	DRD2: dinucleotide (GT) repeat	Individuals who were homozygous for allele 3 were more frequent in the sporadic PD than in controls.	[73]
122 PD patients and 127 healthy controls	DRD4: VNTR 7/48-base pair repeat	No association with PD risk.	[77]
154 PD patients and 125 healthy controls	DRD2: TG/CA repeat (intron 2)	No association with PD risk.	[70]
152 PD patients and 231 healthy controls	DRD2: rs1800497 (2137 C > T) rs1079597 (54716 G > A)	No association with PD risk.	[67]
135 PD patients and 202 healthy controls	DRD2: rs1800497 (2137 C > T) rs1079597 (54716 G > A) rs1801028 (−141 C ins/del) rs1799732 (932 C > G)	rs1800497 (CC genotype) and rs1079597 (GG genotype) were more frequent in PD patients than in healthy controls.	[65]
72 PD patients and 81 healthy controls	DRD2: rs1800497 (2137 C > T)	rs1800497 (CC genotype) was more frequent in PD patients than in healthy controls.	[66]
204 PD patients and 216 healthy controls	DRD2: rs1800497 (2137 C > T) rs1079597 (54716 G > A)	No association with PD risk.	[69]
487 PD patients and 474 healthy controls	DRD1: rs4532 (−48 A > G) rs5330 (150 G > T) rs5331 (595 T > G) rs13306309 (685 G > A) DRD2: rs1799732 (−141 C ins/del) rs1800497 (2137 C > T) rs1800498 (59414 C > T) rs2234689 (72519 C > G) DRD3: rs6280 (25 A > G) rs324026 (32213 G > A) rs1503670 (23948 C > G) rs905568 (g.773 G > A) DRD4: VNTR 120 bp duplication −521 C > T VNTR 7/48-base pair repeat	DRD4 12-bp duplication seems to be associated with PD risk.	[63]
767 PD patients and 1989 healthy controls	DRD2: rs1800497 (2137 C > T) rs1079597 (54716 G > A)	No association with PD risk.	[71]
70 PD patients and 100 healthy controls	DRD2: rs1800497 (2137 C > T) rs1079597 (54716 G > A)	No association with PD risk.	[72]
448 PD patients and 428 healthy controls	DRD3: rs6280 (25 A > G)	No association with PD risk.	[76]
1325 PD patients and 1735 healthy controls	DRD2: rs1800497 (2137 C > T) rs1076563 (55093 T > G) rs6279 (376 C > G) rs6278 (725 G > T) rs273482 (18232490 C > T) DRD3: rs6280 (25 A > G) rs2134655 (65054 G > A)	DRD2 rs1800497 (TT genotype) was associated with increased PD risk in non-Hispanic whites. DRD3 rs6280 (GG genotype) was associated with a decreased PD risk in Hispanics.	[64]
293 PD patients and 369 healthy controls	DRD2: rs1800497 (2137 C > T) DRD4: rs1800955 (4480 T > G)	No association with PD risk.	[68]
4279 PD patients and 5661 healthy controls	DRD2: rs1800497 (2137 C > T) rs1079597 (54716 G > A) rs6279 (376 C > G) rs6278 (725 G > T) rs273482 (18232490 C > T) rs1799732 (−141 C ins/del) rs1076563 (55093 T > G) DRD3: rs6280 (25 A > G) rs2134655 (65054 G > A)	DRD3 rs2134655 and DRD2 rs1800497 were associated with PD risk.	[74]
664 PD patients and 718 healthy controls	DRD2: rs1800497 (2137 C > T) DRD3: rs6280 (25 A > G)	DRD3 rs6280 (genotype CC) was associated with earlier age at onset.	[75]
**PD Motor Symptoms**			
126 PD patients (31 with tremor)	DRD2 rs1799732 (−141 ins/del) DRD2 rs6280	DRD2 −141 ins/del was associated with rigidity.	[80]
591 PD patients (62 with tremor)	DRD3 rs6280 (25 A > G)	No association with tremor in PD.	[78]
**Gastrointestinal Symptoms**			
217 PD patients (56 with GS)	DRD2: rs1799732 (−141 C ins/del) DRD3: rs6280 (25 G > A)	rs1799732 (−141 C ins/ins genotype) and DRD3 rs6280 (GG genotype) was associated with levodopa-induced gastrointestinal symptoms.	[85]
**Impulse Control Disorders (ICD)**			
404 PD patients (58 with ICD) and 559 healthy controls	DRD2: rs1800497 (2137 C > T) DRD3: rs6280 (25 G > A)	DRD3 rs6280 (AA genotype) was associated with ICD.	[95]
89 PD patients (41 with ICDs)	DRD2: rs1800497 (2137 C > T)	No association with ICD.	[93]
91 PD patients (52 with ICD)	DRD1: rs4532 (−48 A > G) rs4867798 (863 A > C) rs265981 (−684 T > A) DRD2: rs1800497 (2137 C > T) DRD3: rs6280 (25 G > A) rs3732783 (51 A > G)	DRD1 rs4532 (AG genotype), rs4867798 (CC genotype) and DRD2 rs1800497 (TT genotype) were associated with ICD increased risk.	[92]
276 PD patients (52 with ICD)	DRD2: rs1800497 (2137 C > T) DRD3: rs6280 (25 G > A)	No association with ICD.	[94]
170 PD patients (70 with ICD) and 285 healthy controls	DRD3: rs6280 (25 G > A)	DRD3 rs6280 (CT genotype) is associated with ICD.	[96]
126 non early-onset PD (NEOPD) and 73 EOPD (age at onset < 45).	DRD3: rs6280 (25 G > A)	rs6280 was associated with ICD in PD patients with an early onset of the disease.	[97]
**Visual Hallucinations (VHs)**			
155 PD patients (84 with VHs)	DRD2 rs1800497 2137 C > T rs1799732 (141 C ins/del) DRD3: rs6280 (25 G > A)	rs1800497 (C allele) was associated with late-onset hallucination.	[104]
88 PD patients (44 with chronic hallucinations)	DRD1 B1/B2 DRD2 rs1801028 (932 C > G) DRD3 1/2 DRD4 7/48-base pair repeat (exon 3)	No association with VHs.	[118]
90 PD patients (45 with VHs)	DRD2: rs1800497 (2137 C > T) DRD3: rs6280 (25 G > A) DRD5: rs6283 (978 T > C)	No association with VHs.	[103]
82 PD patients (42 with VHs)	DRD1: rs4532 (−48 A > G) rs686 (62 G > A) DRD2: rs1800497 2137 C > T rs6277 (957 C > T) DRD3: rs6280 (25 G > A) rs1800828 (−712 G > C) DRD4: rs747302 (−616 C > G) VNTR (7/48-base pair repeat) DRD5: rs6283 (978 T > C)	DRD1 rs686 (AA genotype) was associated with increased VHs risk. DRD1 rs4532 (GG genotype) and rs686 (TT genotype) displayed shorter time to VHs, whereas a longer time to VHs was found in subjects carrying DRD4 rs747302 (CG genotypes).	[18]
**Cognitive Decline**			
134 patients (84 with dementia)	**DRD2** rs6277 (957 C > T)	T/T genotype was associated with increased risk of developing dementia.	[111]
**Sleep Attacks**			
274 PD patients, 137 with sudden onset of sleep (SOS)	**DRD2**: rs1800497 2137 C > T **DRD3**: rs6280 (25 G > A) **DRD4**: VNTR (7/48-base pair repeat)	DRD2 rs1800497 (allele C) was associated with SOS.	[116]
204 PD patients (102 with sleep attacks)	**DRD2**: rs1799732 (141 C ins/del) **DRD2**: rs1800497 (2137 C > T) **DRD3**: rs6280 (25 G > A) **DRD4**: VNTR (7/48-base pair repeat)	DRD4*2 (short) allele was associated with sleep attacks without warning signs.	[117]
**Motor Complications**			
136 PD patients and 224 healthy controls	DRD1 D1.1 in the 5′ untranslated region (UTR), D1.7 in the 39 UTR DRD2 short tandem repeat polymorphism (STRP)	STRP 13- and 14-repetition genotype polymorphism were significantly higher in non-dyskinetic PD patients compared with dyskinetic patients.	[119]
140 PD patients and 140 healthy controls	DRD2: rs1800497 (2137 C > T) DRD3: Bal1, Msp1	DRD2 rs1800497 SNPs may be associated with increased risk for motor fluctuations in PD.	[120]
120 PD patients and 110 healthy controls	DRD5 978 T > C	No association with risk of developing motor fluctuation.	[121]
183 PD patients	DRD2: rs1800497 (2137 C > T) rs1800496 (928 C > T) rs1801028 67518 C > G DRD3: rs6280 (25 A > G) DRD4: VNTR (7/48-base pair repeat)	No association with L-Dopa induced ADR.	[122]
92 PD patients	DRD2 short tandem repeat polymorphism (STRP)	STRP 14- and/or 14/15-repetition genotype was a risk factor for dyskinesia.	[123]
591 PD patients	DRD3: rs6280 (25 A > G)	No association with motor complications.	[124]
503 PD patients and 559 healthy controls	DRD2: rs1800497 (2137 C > T) DRD3: rs6280 (25 A > G)	DRD3 rs6280 (AA genotype) was associated with diphasic dyskinesia.	[125]
199 PD patients	DRD2: rs1800497 (2137 C > T) rs1799732 (−141 C ins/del) rs2283265 (67314 G > T) rs6277 (957 C > T) rs1076560 (63314 G > T) rs2734849 (1472 A > G)	DRD2 rs2283265, rs1076560, rs100497 are associated with dyskinesia.	[126]
352 PD patients	DRD2: rs4245147 (32995 G > A) rs6275 (939 T > C) rs6276 (52 G > A) rs6277 (957 C > T) rs4630328 (16793 C > T) rs17529477 (33935 C > T) rs1079597 (54716 G > A) rs4938017 (47073 G > A) rs4245148 (30583 A > C) rs1079594 (68190 T > A) rs1800497 (2137 C > T)	No association with LID	[127]
100 PD patients (50 with dyskinesia)	DRD1: rs4532 (−48 A > G) rs686 (62 G > A) DRD2: rs1800497 2137 C > T rs6277 (957 C > T) DRD3: rs6280 (25 G > A) rs1800828 (−712 G > C) DRD4: rs747302 (−616 C > G) VNTR (7/48-base pair repeat) DRD5: rs6283 (978 T > C)	DRD3 rs2680 was associated with earlier LID development.	[17]
220 PD patients	DRD2 rs1799732	DRD2 rs1799732 was associated with LIDs.	[128]
228 PD patients	DRD1 rs4532 (−48 A > G) DRD3 rs6280 (25 A > G)	DRD1 rs4532 (G/G genotype) may play a role in occurrence of LID.	[129]
195 PD patients	DRD2 rs1800497 (2137 C > T)	No association with the development of motor fluctuations/dyskinesia.	[130]

**Table 2:** Studies concerning effects of SNPs in DA receptor on response to antiparkinsonian drug therapy.

Approved Indications	Receptor Affinity (ki, nm)	Ddd (g)	Adm.r	Snps	Main Results	Refs.
**Levodopa**						
PD [b]	Dopamine precursor with no intrinsic receptor affinity, which acts indirectly by increasing dopamine levels [b]	3.5 [a]	O [a]	DRD1 rs4532 DRD3 rs6280	DRD1 was associated with occurrence of LID.	[129]
				DRD1 rs4532 DRD2 rs1799732 rs1800497 rs1079597 DRD3 rs6280	No associations between tested SNPs and LID	[147]
				DRD2 rs1799732 rs1801028 DRD3 rs6280	DRD2 rs1799732 and DRD3 rs6280 were associated to occurrence of motor fluctuation after levodopa treatment. DRD2 rs1799732 was associated with time occurrence of LID	[128]
				DRD2 rs1800497	No associations with motor fluctuation or LID	[130]
				DRD1 rs4532 rs686 DRD2 rs1800497 2137 rs6277 DRD3 rs6280 rs1800828 DRD4 rs747302 DRD5 rs6283	DRD3 rs2680 was associated with earlier LID	[17]
				DRD2 rs1799732 DRD3 rs6280	DRD2 rs1799732 and DRD3 rs6280 were associated with an increased prevalence of gastrointestinal symptoms associated with levodopa treatment	[85]
				DRD2: rs4245147 rs6275 rs6276, rs6277 rs4630328 rs17529477 rs1079597 rs4938017 rs4245148 rs1079594 rs1800497	No association with LID	[127]
				DRD3 rs6280 DRD2 rs1800497	DRD3 rs6280 and DRD2 rs1800497 were not associated with LID in Israel and Italian sample of PD	[148]
				DRD2 rs 1076560	DRD2 rs1076560 was not associated with motor sequence learning task and change in manual motor abilities after levodopa treatment	[138]
				DRD2 rs2283265 rs1076560 rs6277, rs1800497, rs2734849	DRD2 rs2283265, rs1076560, rs100497 were associated with LID	[126]
				DRD2 rs1800497 DRD3 rs6280	DRD3 rs6280 (AA genotype) was associated with diphasic, but not pick-dose LID	[125]
				DRD3 rs6280	DRD3 rs6280 did not increase susceptibility to develop levodopa-induced motor complications	[133]
				DRD2 short tandem repeat polymorphism (STRP)	STRP 14 and/or 14/15 repetition genotype was a risk factor for LID	[123]
				DRD2 CAn-STR	13,14 CA repeats was associated with decreased risk to developing peak-dose LID	[146]
				DRD2 rs1800497 rs1800496 rs1801028 DRD3 rs6280 DRD4 VNTR (7/48-base pair repeat)	No association with ADR L-Dopa induced	[122]
				DRD5 rs6283	No association with risk of developing motor fluctuation during levodopa treatment	[121]
				DRD2 STR polymorphism	13 and 14 alleles of the DRD2 STR SNP was correlated with risk reduction of developing peak-dose LID	[119]
**Pergolide**						
PD [b]	D2 (0.2); D3 (0.5); D4 (1.3); D5 (164); D1 (172) Others: 5HT1A (1.9); 5HT2A (8.3); 5HT1D (13.2); 5HT2B (7.1); 5HT1B (281.8); 5HT2C (295.1) [149]	0.003 [a]	O [a]	DRD2 rs1799732, rs1800497 and DRD3 rs6280	D2DR rs1800497 was associated with late-onset VHs, but not with early dopamine-induced VHs	[104]
**Ropinirole**						
PD and RLS [b]	D3 (2.9); D2 (3.7); D4 (7.8), D5 (41,211); D1 (36,600) Others: 5HT1A (288); 5HT1 B (>10,000); 5HT1 D (1380); 5HT2A (>10,000); 5HT2B (3802); 5HT2C (>10,000) [149]	0.006 [a]	O [a]	DRD2 rs1799732, (CA)n STR, rs1800497, DRD3 MscI	The absence of a 15× DRD2 CA repeat allele was related to a decreased discontinuation of ropinirole treatment	[139]
**Pramipexole**						
PD [b]	D3 (0.5); D2 (3.9); D4 (5.1), D5 (>10,000); D1 (>50,000) Others: 5HT1A (692); 5HT1 B (8318); 5HT1 D (1660); 5HT2A (>10,000); 5HT2B (>10,000); 5HT2C (>10,000) [149]	0.0025 [a]	O [a]	DRD3 rs6280	DRD3 rs6280 showed a significant pattern of interaction upon behavioural addiction and higher doses for effective treatment	[97,122,123]
				DRD3 rs6280	DRD3 rs6280 (G/G genotype) require higher pramipexole doses for effective treatment	[134]
				DRD3 rs6280	DRD3 rs6280 (A/A genotype) was associate with response rate to pramipexole	[135]
**Rotigotine**						
PD and RLS [b]	D3 (0.71); D4 (3.9–15); D5 (5.4); D2 (13.5); D1 (83) Others: AR- α1A (176); AR- α1B (273); AR- α2A (338); AR- α2B (27); AR- α2C (135); 5HT1A (30); 5HT7 (86); H1 (330) [150]	0.006 [a]	TD [a]	DRD1 rs4532 DRD3 rs6280	DRD3 rs6280 resulted to increased prevalence for VHs	[151]

Abbreviations. DDD, Defined Daily Dose; ADM.R, administration route; NA: Not Available; O, oral administration, P, parenteral, S-Q, subcutaneous, SL, sublingual, IV, intravenous, TD, transdermal; LID, L-dopa Induced Dyskinesia; VHs, Visual Hallucinations; PD, Parkinson Diseases; ADR, Adverse Drug Reactions; COMT, Catechol-O-methyltransferase; MAO, monoamine oxidase; NMDA, N-methyl-D-aspartate receptor; RLS, restless legs syndrome. [a] https://www.whocc.no (accessed on 25 February 2021); [b] https://www.guidetopharmacology.org/ (accessed on 25 February 2021).
